# Peer support for stroke survivors: a case study

**DOI:** 10.1186/1472-6963-14-256

**Published:** 2014-06-16

**Authors:** Dorothy Kessler, Mary Egan, Lucy-Ann Kubina

**Affiliations:** 1 Bruyère Research Institute, 43 Bruyère St, Ottawa, ON K1N 5C8, Canada; 2 School of Rehabilitation Sciences, University of Ottawa, 451 Smyth Rd, Ottawa, ON K1H 8M5, Canada

**Keywords:** Stroke, Volunteers, Untrained workers, Social participation, Caregivers

## Abstract

**Background:**

Innovative and sustainable programs are required to support the well-being of stroke survivors. Peer support is a potentially low cost way to enhance well-being of recent stroke survivors and the well-being and community reintegration of their peer supporters. This article describes the perceptions of stroke survivors, care partners, peer supporters, and professionals of an individual peer support program.

**Methods:**

An instrumental case study design was used to examine a volunteer peer support program that provides acute care visits and telephone follow-up post-discharge. In particular, a) type of support provided, b) benefits for the stroke survivor and care partner, c) potential harms to the stroke survivor, d) impact of providing support on the peer supporter, and e) required processes were considered. Semi-structured interviews were carried out with 16 new stroke survivors and 8 care partners immediately following hospital discharge and then 6 months later, and with 7 peer supporters, 3 program co-ordinators and 4 health professionals to gather feedback from multiple stakeholders.

**Results:**

Emotional, affirmational and informational support were perceived as being offered by the peer supporters. Peer visits were perceived as providing encouragement, motivation, validation, and decreased feelings of being alone. However, the visits were not perceived as beneficial to all stroke survivors. The impact on the peer supporters included increased social connections, personal growth, enjoyment, and feelings of making a difference in the lives of others. Involvement of the healthcare team, peer supporter training and a skilled coordinator were crucial to the success this program.

**Conclusions:**

Peer support can potentially enhance service to stroke survivors and promote community reintegration for peer volunteers. Further research is needed to determine the preferred format and timing of peer support, and the characteristics of stroke survivors most likely to benefit.

## Background

Stroke is a sudden and disabling event that can affect all aspects of a person’s life [1-3]. The changes and related stigma of stroke can lead to a state of isolation and fear, which can make it difficult to engage in the recovery process [4]. As well, while in hospital, it can be challenging to recognise the importance of information regarding community services; following discharge, when healthcare services may no longer be available, it can be difficult to find information regarding such services. While support from sources such as healthcare professionals, family and friends has been identified as important during post stroke recovery [5], peer support, based on shared experience, may be helpful for decreasing feelings of isolation and fear, and providing information regarding community services.

Peer support is often recommended as a source of emotional, instrumental, informational and affirmational social support for people with chronic conditions [6]. Peer support can offer a unique type of relationship that provides generalization of experience and social validation, and promotes feelings of empowerment [7]. In general, peer support is associated with decreased loneliness and feelings of difference, as well as enhanced social competence, social acceptance, and increased acceptance of chronic conditions [8]. Positive effects have been noted for both the person receiving support and the person providing support [9].

Peer support is a potentially powerful form of support for individuals post stroke. Examples of stroke peer support programs can be found on the web, demonstrating that such programs exist in various settings from acute care to the community and in various formats including one-to-one visits and group social or educational programs. Previous efforts to document the effects of peer support programs have focused on group formats. Ch’ng et al. [10] held multiple focus groups with members of community-based stroke support groups to determine their perspectives on what had helped them recover. Participants listed the stroke group among a number of beneficial psychological interventions. They reported that participation in the group led to feelings of being understood, normalization of their experiences and way of life following stroke, and enjoyment.

Morris & Morris [9] explored the experiences of peer supporters, stroke survivors and care partners who took part in hospital-based peer support groups. Findings revealed the value of peers in the areas of connecting and belonging, and encouragement and empowerment. At least some of all types of participants (that is, in-patient stroke survivors, their care partners and peer supporters) experienced positive feelings associated with being helpful to others through their participation in the groups. Notably, certain aspects of the program (such as open-ended discussion and opportunities for care partners and stroke survivors to discuss issues separately) were experienced negatively by some participants but positively by others. All peer supporters affirmed the importance of thorough training.

Another study used semi-structured interviews to explore the experience of participants in local peer stroke support groups across Canada [11]. Key themes that emerged were that peer support groups: 1) helped stroke survivors understand their stroke in a way that health professionals did not, 2) helped decrease feelings of loneliness or isolation, 3) played a role in overcoming depression 4) provided opportunity for discussion that was different from speaking to family or friends, 5) changed their understanding of the timeframe for improvement and recovery, and 6) inspired a desire to give back and help others.

An evaluation of a group peer stroke support program [12], designed to improve patients’ satisfaction with stroke education and hospital to home transition, identified challenges related to running the program. Group sessions were held on acute stroke wards with information provided by a trained “Peer Role Model” and a healthcare professional. Patient feedback was not included in this evaluation. Peer supporters reported challenges related to their mandate to speak only from their personal experience and expressed interest in further training on communicating appropriately with the in-patient participants. Group scheduling difficulties were noted; that is, scheduled groups were often cancelled when few or no stroke in-patients were available. Also, there was some concern that the one-hour format and long list of topics to be presented were too taxing for the in-patient participants.

While there are clearly benefits of group-based programs, it is also clear that there are challenges to offering such programs in the acute care setting [12]. We were unable to find any evaluations of individual peer support programs for stroke survivors. In an acute care setting, individual peer support programs may better respond to issues related to scheduling group meetings, while allowing for brief sessions and tailoring communication to address specific patient questions or concerns. However, it is not known if individual peer stroke support programs provide benefits similar to those offered by group programs. In this paper we present the results of a case study evaluation of an individual peer support program offered to stroke survivors in an acute care setting. The objectives of this evaluation were to identify:

a) types of support provided,

b) benefits of peer support for stroke survivors and their care partners,

c) harms of peer support for stroke survivors and their care partners,

d) impact of participation on peer supporters, and

e) processes required to offer such a program.

## Methods

A qualitative instrumental case study design [13] was used to conduct an evaluation of the peer support program. A case study can provide valuable information in health services research. This is particularly true in the early phases of investigating a broad question and when a service being examined requires the coordination of a range of stakeholders who may hold different perspectives on this service and its value [14]. The case in this study was the peer support program.

Ethics approval for this study was received from the Bruyère Continuing Care, the Ottawa Hospital and the University of Ottawa Research Ethics Boards.

### Participants

All new stroke survivors and their care partner (when present) who received visits as part of peer support program between February and October 2012 were invited to participate in this study by a peer supporter during the initial visit if the referring health professional judged that they met the inclusion criteria. Criteria for inclusion were: 1) hospitalized on an acute stroke unit following a first stroke, 2) wished to participate in the peer support program and 3) demonstrated adequate cognitive and communication skills to participate in qualitative interviews (with or without supported communication techniques). In addition, all peer supporters and coordinators who were involved in the peer support program, and healthcare professionals who worked on the hospital unit where the program was offered were informed about the project by the researchers during a scheduled meeting and invited to contact the researchers if they were interested in participating. The goal of sampling was to gather information and feedback from all groups of stakeholders and, in doing so, achieve multiple perspectives on the process and outcomes of the program.

Written informed consent to take part in this study was obtained from all participants.

### Program description

The Stroke Survivors Association of Ottawa (SSAO) offers a peer support program and post discharge telephone follow-up support to people receiving care on an acute stroke unit. The objective of the program is to ensure that patients have the opportunity to meet with a stroke survivor who is experiencing good community reintegration. The goals of this program are to provide hope to newly diagnosed stroke survivors, decrease feelings of stigma and isolation, and offer information about post-stroke community services. The initial visit is designed to provide a message of hope; the length of this visit is approximately ten minutes. Subsequent post-discharge telephone visits are made to the stroke survivor or care partner at one, three, six, nine and twelve months. The objective of these calls is to provide on-going support, as well as information related to living with stroke and community resources. The length of these telephone visits varies between 5 and 60 minutes.

The Executive Director of SSAO (a paid position) acts as the Program Coordinator. She is supported by two volunteer program coordinators who are stroke survivors. These volunteer program coordinators are responsible for collecting post discharge contact information for the new stroke survivors (Logistics Coordinator) and scheduling of on-site visits (Visit Coordinator).

All three coordinators are involved in the training of the Peer Supporters. Training consists of a combination of six hours of in-class group education provided by the Executive Director and Logistics Coordinator, a two hour orientation to the hospital, and four to six individual sessions shadowing the Visit Coordinator as she carries out peer support visits. In-class training covers procedures, roles and appropriate topics of discussion, while shadowing provides opportunity for practice of routines and development of communication skills through modelling and feedback.

Referral for peer support is coordinated by two designated staff on the acute stroke unit who are also members of the Regional Stroke Team. One staff member approaches each patient who meets the referral criteria and completes a referral sheet. Stroke survivors with mild to moderate deficits and basic cognitive and communication skills are referred for peer support. Patients are excluded if they have more severe deficits, are medically unstable, have global aphasia, or have a planned discharged to long-term care. Visits occur one day per week. On the day of the visits, the Visit Coordinator picks up the referrals and assigns peer supporters to each patient. The peer supporters conduct their visits working in pairs, these pairs being matched to complement each others’ strengths and degree of disability.

Follow-up telephone calls are then organized by the Logistics Coordinator who tracks information and sends reminders to those scheduled to do the visits. These follow-up calls had initially been made by a paid staff member, but when the funding for this position was no longer available, peer supporters were asked to make follow-up phone calls to new stroke survivors they had visited in hospital.

In 2012, the program provided acute care visits to 112 stroke survivors and provided at least one follow-up phone call to 82% of the new stroke survivors previously or their care partners.

### Data collection

Open-ended, semi-structured interviews were carried out with new stroke survivors and their care partners, peer supporters, coordinators and health-care professionals. The interview questions focused on the type of support provided through the visits, perceived impact of the program, aspects of the program found to be particularly helpful, and areas for development. Interviews with new stroke survivors and care partners were carried out shortly after discharge from acute care and six months later. Interviews with all other participants were carried out at one point in time during the study. Interviews were recorded and transcribed for analysis. In addition, peer supporters kept diaries following in-person hospital visits and post discharge follow-up phone calls. In the diaries they recorded the content of each visit along with their thoughts and feelings about each visit. Recruitment and data collection continued over a one year period at which time analysis revealed that no new codes were emerging and saturation had been reached.

Demographic data and information related to severity and location of stroke were collected from stroke survivors and peer supporters at their entry into the project. As the two coordinators were also stroke survivors who conducted peer visits, their demographic information is included with those of the other peer supporters. For care partners, information on relationship to stroke survivor, living situation and work status was gathered. Data on years of experience were collected from health professionals. The team on the acute stroke unit consisted, for the most part, of one person per health profession. Because identification of the profession could effectively identify some participants, data on the profession of the health care providers are not reported.

### Data analysis

MacPherson and McKie’s [15] recommendations for the use of qualitative data in program evaluation were followed. First, six interview transcripts and diaries were read by the primary investigator and research assistant to gain a sense of the data. Then, working separately, the primary investigator and research assistant created a coding scheme using the first two participants from each of the following groups: new stroke survivors, peer supporters, program coordinators and health professionals. The primary investigator and research assistant then met to compare coding and refine the coding schemes. Consensus was used to resolve any discrepancies related to coding. Coding schemes were then applied to all transcripts by the research assistant. Following this, coded units were entered in a preliminary analytic grid that was used to organize information critical to the research objectives. The grid was designed to organize codes for each objective according to participant type. It initially included types of support, benefits to stroke survivors, harms to stroke survivors and impact on peer supporters. The grid was later expanded to include challenges faced in relation to different aspects of the program and strategies to overcome challenges. Finally, data from this grid were summarized according to the research objectives. The qualitative research software Atlas.ti [16] was used to aid in the organization of the data. Documents for each stage of analysis were kept to create an audit trail. Illustrative quotes from one participant were translated from the original French for inclusion in this paper.

## Results

Of 87 new stroke survivors who were invited to participate, 54 expressed interest. Of these, 31 could be contacted and 17 consented to participate. One person could not be contacted after signing the consent form. Sixteen new stroke survivors completed initial interviews, and fourteen completed the 6-month interview (one could not be contacted and one declined to be interviewed due to other health issues). Six care partners also took part in the initial interview and eight took part in follow-up interviews. All interviews with stroke survivors and care partners were carried out together, at the participants’ request. Seven of the thirteen peer supporters, all three program coordinators and four health professionals who identified themselves as being familiar with the program were also recruited into the study. These health professionals were representative of the various team members on the unit.

Demographic information for the new stroke survivors and peer supporters is presented in Table 1. Of the eight care partners, seven were women, all were spouses living with the stroke survivor, six were retired and two were working. The health professionals had an average of 21 years of experience (range: 17–26 years).

**Table 1:** Participant demographic information

	**Stroke survivors (N = 16)**	**Peer supporters (N = 7)**
Mean age in years (SD), range	64.8 (11.3) 35-81	59.3 (9.6) 47-72
Number of men (%)	12 (75.0%)	4 (57.1%)
Stroke type (%): Clot	14 (87.5%)	3 (42.9%)
Bleed	2 (12.5%)	4 (57.1%)
Stroke location (%): Left	3 (18.8%)	2 (28.6%)
Right	13 (81.3%)	5 (71.4%)
Mean Barthel Index score (SD), range	78.8 (21.7)	95.0 (7)
	40-100	85-100
Living situation (%): Live alone	4 (25.0%)	2 (28.6%)
Live with spouse	10 (62.5%)	3 (42.9%)
Other family member	2 (12.5%)	2 (28.6%)
Supports (%): Personal care	7 (43.8%)	0 (0%)
Therapy	9 (56.3%)	1 (14.3%)
Transportation	2 (12.5%)	2 (28.6%)
Other	3 (18.8%)	3 (42.9%)

Data collection took place over a period of 10 months. A total of 17 acute care visit diaries (5 peer supporters, 2 volunteer program coordinators) were collected. As well, post-discharge telephone contact diary records were obtained for 28 stroke survivors. Follow-up telephone calls were primarily completed by the paid Program Coordinator with a few completed by peer supporters and a volunteer coordinator. This change from the original plan was put in place when it became clear that the time commitment, organizational and communication skills and knowledge of resources required for these calls to be effective necessitated that they be carried out by the Program Coordinator.

Results are presented below according to each research objective. The sources of data for each of the information categories are presented in Table 2. Pseudonyms have been assigned to participants for reporting purposes.

**Table 2:** Sources of data

	**Stroke survivor**	**Care partner**	**Peer supporter**	**Healthcare professional**	**Program coordinator**
Type of support	X	X	X	X	X
Benefits to stroke survivors	X	X	X	X	X
Harms to stroke survivors	X	X		X	X
Impact on peer supporters			X		X
Challenges	X	X	X	X	X

### Type of support provided

Data collected from the diaries and interviews with the new stroke survivors and peer supporters identified that emotional support was provided during the initial in-hospital visit in the form of hope, encouragement, and reassurance. Emotional and affirmational support occurred through taking time to listen, sharing of stories and validation of feelings.

I honestly didn't want them to leave because I just wanted to continue talking to someone who actually had gone through what I went through. (New Stroke Survivor-Sylvie, initial interview)

Informational support was also offered during the initial and telephone visits. Peer supporters provided written as well as verbal information on resources in the community including those offered by SSAO. During the initial visit this information tended to be more general in nature. However, during follow-up telephone calls, information was targeted to the new stroke survivor’s or care partner’s needs and included information on specific services available in the community. Information to assist with both finding and accessing services was provided to six out of the 28 people for whom follow-up diaries were kept.

### Benefits of peer support for the stroke survivors and their care partners

All groups identified benefits for the new stroke survivors and their care partners. The emotional support provided was seen as beneficial at a time when stroke survivors and care partners were feeling overwhelmed by the unknown. The visit from the peer supporters encouraged and motivated the stroke survivors to work towards recovery. After having someone who had gone through a similar experience take time, listen, share experiences, and make a connection, stroke survivors reported feeling validated and less alone. Information received from a peer regarding the experience of living with stroke was generally given more value than that received from a healthcare professional.

And they [peer supporters] provided sort of reassurance… and provided a real face. When you're dealing with doctors and nurses they're great…. But they're medical people and they can talk to you about what you've just gone through, but the chances are more than likely that they haven't… The people in Stroke Survivors, well they have. (New Stroke Survivor-Mike, initial interview)

Peer supporters were also seen as a source of inspiration by some stroke survivors who expressed an interest in pursuing a peer supporter role in the future.

Care partners also benefitted from the emotional support. They reported feelings of reassurance and decreased isolation, particularly from the follow-up phone calls.

It reassures me, you know, they ask “How is [name of partner]? Can we do anything for you?” This is very important. (Care Partner-Liz, 6 month interview)

A few care partners reported that they had read the information kit and found it helpful.

But it’s nice to have all those resources [in the SSAO information kit] that you can contact because you’re overwhelmed sort of, the person is really ill and you’re wondering what are they going to need. (Care Partner-Sue, initial interview)

### Harms of peer support for the stroke survivors and their care partners

No specific harms of the visits were identified by the new stroke survivors. However, potential harms were identified by one care partner, one health professional and one coordinator. The care partner felt that her partner was not ready to receive this type of visit because his condition had not stabilized enough for the message of hope to be perceived as realistic.

Yeah I thought it was maybe a little bit premature … He wasn’t sleeping well, he wasn’t medically stable… he wasn’t really ready for someone to tell him everything was going to be all right because it wasn’t all right. (Care Partner-Joanne, initial interview)

It is interesting to note that the new stroke survivor in this situation did not voice such concerns.

The health professional raised concern about potential harms when patients with mild stroke were visited by peer supporters with more obvious physical disabilities. This health professional and one of the coordinators reported receiving feedback from a few new stroke survivors that being visited by someone with a significant visible disability (such as someone using a wheelchair) was upsetting. The Program Coordinator reported that she had received calls to SSAO expressing this concern.

Two calls … from people that we visited on the acute care floor [informed us] that they did not appreciate a person in a wheelchair coming in for the visit, …And to have somebody come in in a wheelchair could actually make them feel as if they're not going to be able to walk again. (Coordinator-Carol)

New stroke survivors and healthcare professionals also expressed concern about the number of peer visitors present during a visit. When there is a new peer supporter being trained the number of peer supporters may increase from two to three. Feedback indicated that one-on-one interactions were preferable, having two peer supporters visit was acceptable, but any more could be overwhelming for the stroke survivor.

### Impact of peer role on the peer supporter

All peer supporters and coordinators indicated that offering support to fellow stroke survivors was beneficial to the peer supporter. While concern was expressed by program coordinators about the potential emotional impact on peer supporters for whom the visit may trigger past feelings related to the initial experience of stroke, none of the peer supporters interviewed described experiencing these types of feelings. A few peer supporters reported concern about their ability to do a good job and be understood, particularly with new stroke survivors who had more severe impairments or significant aphasia.

I’m not always comfortable going into a room maybe if somebody has really severe disability, and if they can’t talk or they’re really upset or… I don’t know what else to say. (Peer Supporter-Charles)

Some people you go and see, they’re so deeply depressed, and some of them that has aphasia that can’t speak to you, it makes me feel real sad. (Peer Supporter-Steve)

Another concern expressed by one peer supporter was the feeling that since he had no visible disability, he may lack credibility among new stroke survivors who did not believe that he had experienced a stroke.

Another, more frequent concern expressed by peer supporters was their ability to remember procedures and routines. Due to the fact that peer supporters may only be scheduled once per month, some peer supporters reported feeling “bothered” about their ability to remember required details.

For me like six weeks is like forever and I just forget the whole, I forget all my training … Not all my training but important parts. (Peer Supporter-Charles)

Peer supporters who used the local adapted transportation service had the additional frustration of sometimes arriving late and missing the visits.

Personal benefits noted by peer supporters included increased social connections, personal growth, enjoyment and the feeling that they had been able to make a difference in the lives of others. Following the weekly visits, the peer supporters would go for coffee which provided an opportunity for social interaction, support, and peer mentoring. Several peer supporters reported that while the visits posed challenges mentioned above, they were able to push themselves, and build coping skills and confidence, all of which contributed to their personal growth. Positive feedback received from new stroke survivors reinforced a sense of purpose - a sense that they had contributed.

### Perceived processes required to offer such a program

The processes involved in setting up the peer support program involved close collaboration with the healthcare team to negotiate the referral process, the type of information to be provided to stroke survivors by the peer supporters, and duration of visits. Processes were put in place to ensure that privacy and confidentiality of patient information were protected, and that safety concerns for peer visitors were addressed. An important facilitator for the peer supporters was that parking costs were covered by the hospital.

Commitment on the part of healthcare professionals who were willing to take the time to complete the referrals despite busy caseloads was a key program component. These professionals also provided important on-going collaboration to address program challenges and improve service to stroke survivors. While data was being collected both the referring professionals and the program coordinators expressed an interest in developing feedback mechanisms following initial visits. Two important goals for these mechanisms were to ensure that all eligible patients were seen, and to ensure health professionals were aware of any issues brought up during the visits that they could help address.

The recruitment, training and orientation of the peer supporters were also identified as critical to program success. Training provided coordinators with a method of ensuring all peer supporters were aware of the program mandate and procedures. The peer supporters also noted that the training provided them with an opportunity to develop communication skills and start building confidence in their new roles.

At first it was new … I didn’t know if I was going to be able to do it [volunteer role], to feel like I could contribute and then I learned that yes I could, that it was good, and that gave me the courage I needed to keep on [volunteering]. (Peer Supporter-Michelle)

Common preferred characteristics of peer supporters were identified by all categories of participants. These were being authentic, friendly, confident, a good listener, knowledgeable regarding resources and programs and respectful of the stroke survivor. As noted earlier, visibility of the peer supporter’s disability had a negative impact on some stroke survivors, but promoted the feeling of shared experience for others.

The support offered via telephone calls post discharge from hospital was felt by program coordinators and peers supporters to be an important resource for stroke survivors. However, these follow-up calls posed many challenges. Primary among these was the ability to reach people once they left hospital. Although the coordinators reported reaching 82% of the stroke survivors whom they visited in hospital, they felt that the people they could not contact may have been those most in need of support. Additionally, while it was originally hoped that the peer supporter who visited the stroke survivor in hospital would complete the follow-up call, many of the peer supporters did not feel that they had the knowledge and skills to identify and provide the needed support once stroke survivors left the hospital. As well, the organizational skills required to track calls until each individual was reached were a challenge for peer supporters with cognitive deficits. Peer supporters noted that organizing call-backs took a lot of effort and that it was difficult to stay on top of information regarding available services. Coordinators noted the need to remind peer supporters to make calls. For this reason, the majority of telephone follow-up visits were completed by the Program Coordinator.

## Discussion

This pilot evaluation revealed several perceived benefits for new stroke survivors and their care partners. The peer support program offered emotional, affirmational, and informational support. The emotional and affirmational support instilled hope and feelings of validation and decreased a sense of isolation. In a conceptual analysis of peer support, Dennis [6] related the exchange of emotional support to feeling accepted, respected, and valued despite personal difficulties, and affirmational support to positive future expectations. Stewart and colleagues [17] identified similar types of support provided to caregivers of individuals with stroke which reinforced self-esteem and confidence. Informational support varied from provision of an information package to assistance navigating resources in the community. Navigation support, that is help locating and accessing services, has been identified as a need for community-dwelling stroke survivors and their carers [18]. The benefits of emotional and affirmational support have been identified in evaluations of group peer support programs [9-11]. However, the benefit of informational support was not clearly identified in the evaluations of group peer support. Our study demonstrates that individualized peer support programs may offer similar benefits to group programs while also addressing informational needs. Stroke survivors consistently report difficulties meeting informational needs [19]. Individual peer support may provide a promising avenue to address these needs.

In addition to potentially providing a higher level of informational support than is available in other programs, individual peer support may help overcome the logistic challenges of group peer support. These include transportation and scheduling issues [12,20].

Previous studies of peer support have not consistently examined potential negative consequences [6]. In this study we noted that, along with the above benefits, the potential for the initial visit to increase new stroke survivor distress was identified. While none of the new stroke survivors interviewed reported being distressed by peer visits in hospital, a care partner did feel that these visits offered unrealistic hope. As well, during the evaluation, the organization did report calls to the office from new stroke survivors who expressed that they had been upset by visits. It was not clear whether the feedback received by the health professional was initiated by concerns of patients or their family members. It may be that some stroke survivors interviewed were distressed by the visits but were reluctant to provide negative feedback.

This evaluation revealed important benefits for the peer supporters. These benefits included increased confidence, a sense of contributing, personal growth and increased social connections. One evaluation of a stroke peer support group program reported a similar benefit for peer supporters of positive feelings associated with helping others [9]. As well, similar results were seen in a study of individualized peer support of persons diagnosed with multiple sclerosis. Here, peer supporters reported improvements in confidence, self-awareness, self-esteem, mood and role functioning [21]. Opportunities for meaningful social participation have been identified as an important need of stroke survivors [22]. Peer support programs appear to be one effective way of filling this need.

This evaluation identified several processes that were important for the success of the program, as well as some challenges. Participants noted their appreciation of the training that they received and the support and fellowship provided when several peer supporters were present on the unit at the same time. The structure of the program was such that it provided a relatively safe environment to face the challenges posed by the peer supporter role. Support and mentoring provided by coordinators and other peer supporters helped to create this safe environment.

Collaboration between the coordinators and healthcare professionals also appeared to be a key ingredient of the program. Both groups felt that formal processes to promote on-going information sharing and collaboration would be beneficial for on-going program success and development. For example, access to the peer support program could be improved through feedback processes to ensure that all new stroke survivors receive a visit and that information required for follow-up is accurate and current. As well, discussions between these groups could include ways to address the needs of stroke survivors not currently receiving visits, such as those with a planned discharge to long-term care.

Initially, it was hoped that the peer supporters could provide telephone follow-up to the new stroke survivors they had met in hospital. However, this work proved to be too challenging, in terms of organization, communication and knowledge of services. This study demonstrated that such follow-up services may require skills and knowledge beyond what a volunteer peer supporter can typically provide.

To our knowledge, this is the first formal evaluation of an individualized peer support program for stroke survivors. Based on the results of this study, individualized peer support programs can be considered a potentially low cost way to enhance services provided by healthcare professionals. Social isolation and problems with community integration are well-known challenges for stroke survivors [23]. Peer support may play a role in decreasing social isolation and enhancing community reintegration, particularly among those providing peer support.

### Limitations

This research examined one peer support program as a single case. Findings may not be generalizable to other peer support programs. Data collection relied on interviews with stroke survivors a few weeks after the initial visit and six months later. The initial delay may have altered the way in which they viewed the visit.

Stroke survivor participants included only those patients on the unit who agreed to both a peer visit and taking part in the study. As a result, they were potentially more representative of people who are likely to view such a visit positively. As well, the initial referral criteria for the peer support program excluded stroke survivors who would be going to long-term care. Therefore the potential impact of peer support on this group of stroke survivors was not included in this evaluation.

Finally, this study was carried out at the request of the agency providing this service. While members of the research team do not have any direct affiliation with SSAO, two have worked with the agency on previous evaluation projects and therefore do have a relationship with the agency and some of its employees. While the research team made a conscious effort to conduct the analysis and present findings in an objective manner, the findings may be biased towards more positive outcomes as a result of this relationship.

## Conclusion

This study provides a description of the experience of stroke survivors, their care partners, and peer supporters who took part in an individualized peer support program during the course of the study and provides an understanding of the range of benefits arising from peer support, the possibility of negative outcomes, and the processes required to offer such a service. While this peer support program is just one case of peer support for stroke survivors, this examination advances the understanding of this type of service in general.

Peer support programs for stroke survivors are emerging provincially, nationally and internationally. These programs have the potential to provide much needed support to stroke survivors and to promote well-being of peer supporters. However, further evaluations of these programs are needed. Potential questions that should be address in future studies are: 1) what are the characteristics of stroke survivors and care partners who may benefit from individual peer support, 2) when is individual peer support indicated over group programs and 3) what are the long-term impacts of peer support programs on stroke survivors, care partners and peer supporters.

## Abbreviations

SSAO: Stroke Survivors Association of Ottawa.

## Competing interests

The authors declare that they have no competing interests.

## Authors’ contributions

Study design - DK, ME, LK. Supervision of research project – DK. Data collection, - LK. Data analysis and interpretation; initial manuscript preparation – DK, LK. Critical review of manuscript - ME. All authors read and approved the final manuscript.

## Pre-publication history

The pre-publication history for this paper can be accessed here:

http://www.biomedcentral.com/1472-6963/14/256/prepub
